# Validity and reliability of ultrasonographic assessment of femoral and tibial torsion in children and adolescents: a systematic review

**DOI:** 10.1007/s00431-024-05619-y

**Published:** 2024-06-03

**Authors:** Xavier Ruiz-Tarrazo, Carles Escalona-Marfil, Gil Pla-Campas, Andrea Coda

**Affiliations:** 1 Faculty of Health Sciences at Manresa, University of Vic - Central University of Catalonia (UVic-UCC), Av. Universitària, 4-6, 08242 Manresa, Spain; 2 University of Girona, Girona, Spain; 3 Faculty of Education, Translation, Sport and Psychology at Vic, University of Vic - Central University of Catalonia (UVic-UCC), C/ Sagrada Família 7, 080500 Vic, Spain; 4 Podiatry, School of Health Sciences, College of Health, Medicine and Wellbeing - The University of Newcastle, Newcastle, Australia; 5 Equity in Health and Wellbeing Research Program, Hunter Medical Research Institute (HMRI), New Lambton Heights, New South Wales, Australia

**Keywords:** Femoral antetorsion, Femoral anteversion, Tibial torsion, Ultrasonography, Echography, Children

## Abstract

Torsional disorders of the lower limb are common in childhood, and they are one of the primary reasons parents seek consultation with healthcare providers. While clinical manoeuvres can assess femoral and tibial torsion, their reliability is medium to low. Various imaging-based techniques, including computed tomography, magnetic resonance imaging, fluoroscopy, biplanar radiology and ultrasonography, have been used to evaluate torsional alterations of the lower extremity. Among these, ultrasound assessment offers certain advantages: it is a low-cost, non-irradiating technique, which allows the follow-up of children’s torsional development. However, to the best of the authors’ knowledge, its validity and reliability have not been summarised in a systematic review. This study aims to analyse the validity and reliability of ultrasonography in determining femoral and tibial torsion in children and adolescents. A search from Medline (via PubMed), Web of Science, Scopus and CINAHL databases were performed from inception to 16 March 2023. No restrictions were placed on the publication year or language. The methodological quality of all eligible studies was independently reviewed by two authors using QUADAS and STARD checklists. Overall, 1546 articles were identified through the searches; 30 were considered eligible for full-text screening; and 8 studies were finally included in this review. The included studies were conducted in Germany, Norway and the UK. Among them, 7 studies analysed the validity of ultrasonography compared with other imaging techniques such as computed tomography, magnetic resonance imaging and biplanar X-ray, and 4 studies assessed intra- and inter-observer reliability. All the studies assessed femoral torsion, but only one of them also included tibial torsion.

*     Conclusion*: Ultrasound is a good alternative for routine evaluation and follow-up of femoral torsional alterations in children and adolescents due to its safety, accessibility and immediate results in the clinical examination room. Although ultrasound has good accuracy and reliability for routine evaluations, there is controversy about whether it is sufficient for surgical planning. In cases where greater accuracy is required, magnetic resonance imaging and biplanar radiography are the preferred imaging techniques.

**Table:** 

**What is Known:** *• Several imaging-based techniques have been described for the assessment of torsional alterations of the lower extremity.* *• Computed tomography, magnetic resonance imaging, biplanar radiology and ultrasonography are the most used and studied methods.*	
**What is New:** *• Ultrasonography represents a good alternative for the assessment of femoral and tibial torsional alterations in children and adolescents, given its safety, accessibility and immediacy of results in the consultation room.* *• Its accuracy and reliability are good but not sufficient for surgical planning, in which case MRI and biplanar X-ray will be the preferred choices.*

**What is Known:**

*• Several imaging-based techniques have been described for the assessment of torsional alterations of the lower extremity.*

*• Computed tomography, magnetic resonance imaging, biplanar radiology and ultrasonography are the most used and studied methods.*

**What is New:**

*• Ultrasonography represents a good alternative for the assessment of femoral and tibial torsional alterations in children and adolescents, given its safety, accessibility and immediacy of results in the consultation room.*

*• Its accuracy and reliability are good but not sufficient for surgical planning, in which case MRI and biplanar X-ray will be the preferred choices.*

## Introduction

Torsional disorders of the lower limb refer to a group of conditions characterised by abnormal twisting of the leg bones, typically the femur or tibia. These disorders are a common source of concern for parents and a leading reason for paediatric consultations with healthcare providers. As many as 10% of the paediatric population may present with a torsional disorder [1]. The most common clinical manifestation is an in-toeing gait, whose clinical evolution is often benign and self-resolving. However, in some cases, it can lead to clumsiness, difficulty in walking or cosmetic disturbances [2], and in certain instances, they can alter the biomechanics of gait and cause pathologies such as patellofemoral arthritis and hip dysplasia, among others [3–6].

The factors that determine the torsional profile of the lower limbs are mainly femoral torsion (FT), and tibial torsion (TT) [7]. These parameters typically stabilize by the age of 9–10 years as the bones mature and rotate externally [8, 9].

Clinical assessment of FT and TT involves various methods, with differing degrees of reliability. FT is commonly measured with the Craig test, also known as the trochanteric prominence angle test, where the external palpation of the greater trochanter is used to assess the rotation [10–12]. TT can be evaluated using the posterior surface of the tibial condyles and the transmalleolar axis as reference points. Although these tests can be useful in a clinical setting, their reliability is medium to low [13–15] and are not suitable for accurately monitoring torsions during childhood [11, 13, 15–17].

Alternatively, imaging tests represent the most accurate way to quantify femoral and tibial torsion [12, 18–24]. Different imaging-based techniques have been proposed, including computed tomography (CT), magnetic resonance imaging (MRI), fluoroscopy, biplanar radiology and ultrasonography (US).

CT has traditionally been the “gold standard” for assessing torsional deformities due to its high accuracy and reliability [25, 26]. However, the use of CT is limited by its cost, availability and radiation exposure, making it less suitable for repeated examinations, especially in children [27, 28].

MRI is a highly precise and non-radiating technique [25, 29–31], but there is limited data on the reliability and reproducibility of MRI-based torsional measurements [29]. Additionally, the interchangeability of MRI and CT for measuring femoral torsion remains controversial, with both methods being costly and time-consuming [32].

Low-dose stereoscopic X-ray with 3D reconstruction is a fast and accurate alternative, providing a full-body biplanar X-ray in less than 20 s [33, 34]. Its use is increasing in paediatrics because the X-ray exposure is 800 to 1000 times lower than that of CT, and it allows an accurate and safer follow-up of the torsional parameters of the lower extremities [24, 34–36]. However, its availability in clinical practice is limited due to a high cost and, although lower than CT, it is still a radiating technique.

US is a non-radiating, reliable and accurate alternative for the evaluation of torsional alterations of the lower limb [14, 21, 22, 37–40], and it is less expensive and less time-consuming compared to CT and MRI. Furthermore, it can be performed in the clinical setting, as it does not require a specialised environment, unlike CT, MRI or X-ray [16, 22]. Different protocols have been described to quantify the femoral and tibial torsion using various anatomical references, showing good reliability in the adult population [22, 37, 41].

Although US may serve as a viable non-radiating alternative for the assessment and monitoring of torsional parameters in children [10, 37], few studies assess femoral and tibial torsion in children, and no systematic review has been previously made on their validity and reliability.

## Objective

This systematic review aims to analyse the validity and reliability of ultrasonography for quantifying femoral and tibial torsion in the paediatric and adolescent population. A second objective is to describe the different anatomical references used for the assessment.

## Methodology

This systematic review was conducted according to the Preferred Reporting Items for Systematic Reviews and Meta-analyses (PRISMA) guidelines [42]. The protocol was registered in the Prospective Register of Systematic Reviews (PROSPERO) database before starting the database searches (ID: CRD42021290973).

### Search strategy

Medline (via PubMed), Web of Science, Scopus and CINAHL databases were searched based on their broad coverage and reputable quality in medical and health-related research. The search is performed from each data-base’s inception to March 2023, using the terms and strategy presented in Table 1. No restrictions were imposed on publication year or language.

**Table 1:** Terms and search strategy in the different databases

1. Femur OR femoral OR tibia* OR lower limb * OR lower extremity*
2. Version OR anteversion OR torsion* OR antetorsion*
3. #1 AND #2
4. Ultraso* OR Ecography OR Imag*
5. #3 AND #4

* = truncate

### Eligibility criteria

Studies evaluating the validity and/or reliability of ultrasonography for the assessment of femoral and/or tibial torsion in children and adolescents under 18 years of age were included.

Studies in neurologically impaired populations, in artificial or cadaveric models and in animals were excluded.

### Selection of studies

Two authors (XR and AC) independently screened the titles, abstracts and full texts of articles based on inclusion and exclusion criteria. Any discrepancies were resolved by discussion between the two reviewers or by a third author (CE) until consensus was reached.

### Data extraction

Data extraction was performed using a standardised template and included: lead author of the study, year of publication, country, participant demographics (sample size, sex and age), intervention characteristics and validity and/or reliability results of the methodology under study (Table 3).

### Risk of bias assessment

The methodological quality of all eligible studies was independently reviewed by two authors (XR and GP). Standards for Reporting Studies of Diagnostic Accuracy (STARD) [43] and Quality Assessment of Diagnostic Accuracy Studies (QUADAS) [44] were used according to the protocol proposed by Fernandes De Oliveira et al. [45]. Each criterion was assigned with a judgement of ‘yes’, ‘no’ or ‘unclear’. Any disagreement was discussed until consensus was reached or resolved by a third author (CE).

### Data analysis

Descriptive statistics (frequency, mean, range and percentage) were used to characterise the participant population and the intervention of the included studies. No meta-analysis could be conducted due to the heterogeneity of data obtained.

The validity is assessed by examining the study design, statistical measures and comparisons with a recognised gold standard [46]. The reliability is evaluated in terms of intra- and inter-observer consistency, using statistical measures to determine agreement among different observers and across repeated trials [46].

## Results

**Fig. 1:**
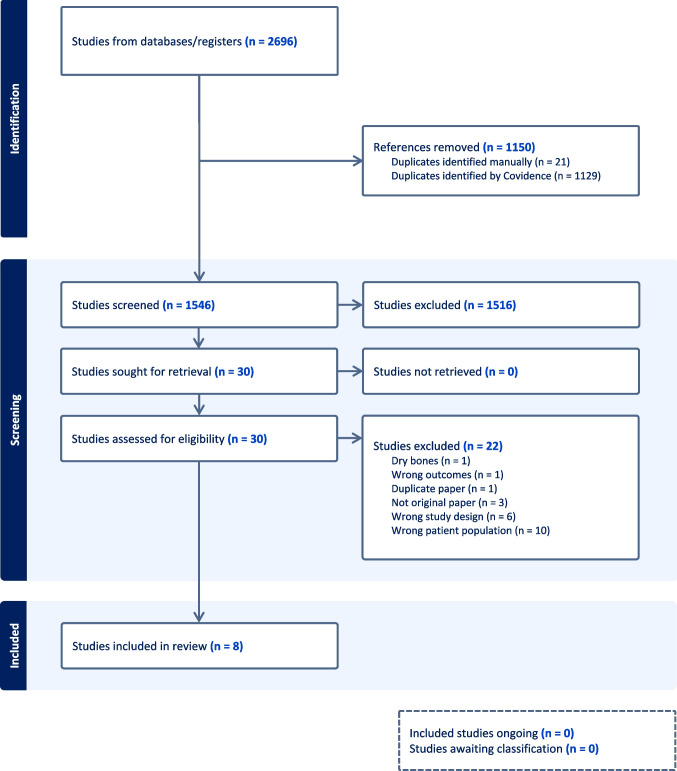
Flowchart for the selection of the studies

Out of 1546 articles identified through the search, 30 articles were considered eligible for full-text screening, and 8 studies were eligible for inclusion in this review (Fig. 1). Using the QUADAS & STARD protocols to evaluate methodological quality [43, 44], five of the eight reviewed articles scored 10 or more ‘yes’ responses on the 15 criteria analysed. Two articles scored between 5 and 10, while only one article scored below 5. This article aimed to analyse the reliability but not the validity against a gold standard (Table 2).

**Table 2:**
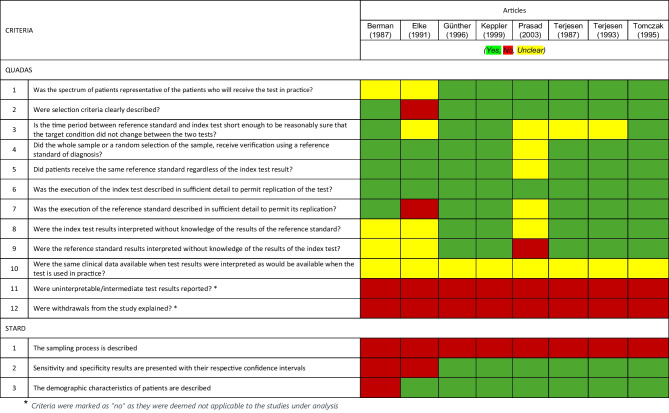
Results of the assessment based on the 15 criteria, 12 from QUADAS [43] and 3 from STARD [42] for the 8 articles included in this systematic review.

A total of 286 participants were included, and 566 lower limbs were assessed. As for the gender of the participants, excluding Berman et al. [47] which does not provide this data, differences are small, with an average of 51.2% of females. The age range is very similar in 6 of the 8 studies (from 2 to 17 years) with a mean of 8.4 years. Berman et al. [47] does not provide data, and Prasad et al. [48] assessed infants between 0 and 6 months of age. Three studies (both studies from Terjesen [38, 49] and Elke et al.’s [50]) include children and pre-adolescents (3–14 years), while those of Gunther et al. [51], Keppler et al. [52] and Tomczak et al. [53] also include adolescents (2 to 17.5 years old). All participants were recruited from children’s hospital clinics. The main characteristics of the included studies are summarised in Table 3.

**Table 3:** Characteristics of eligible studies included in this systematic review

**Author (year) country**	**Participants**					Analysis	
	*n*/limbs	Mean age (range) % of girls	Characteristics	Origin	Structure analyzed	Validity vs	Reliability
**Berman et al. **[47]** UK**	10/19	Not informed	1, only 1 hip for pelvic obliquity 6, pre-SG CT for juvenile chronic arthritis 3, normal. Reason for CT not reported	Northwick Park Hospital, Harrow	Femur	CT	-
**Elke et al. **[50]** Germany**	29/55	6.9 (3–11) 48%	Not informed	University Clinic Basel	Femur	X-ray	Inter-observer (3-observers)
**Günther et al. **[51]** Germany**	21/41	11.1 (3.2–17.5) 38%	5, SG derotator planning by CP 7, Hip maturation disorders 5, Perthes disease 4, Idopat or post-trauma rotational Alt	Orthopaedic Clinic of the University of Ulm	Femur	CT, MRI and GM	Intra-observer Inter-observer (*2 observers*)
**Keppler et al. **[52]** Germany**	50/100	8 (2–16) 30%	50, follow-up for femur or tibia fracture	Safranberg Surgical Clinic, University of Ulm	Femur and tibia	CT	-
**Prasad et al. **[48]** UK**	37/74	10.4 weeks (2–28) 59%	37, routine examination for suspected or risk factors for developmental dysplasia of the hip	Royal Liverpool Children’s Hospital	Femur	-	Intra-observer Inter-observer (*2 observers*)
**Terjesen and Anda **[49]** Norway**	57	7 (3–14) 82%	Most participants admitted for examination for clinical signs of femoral anteversion	Trondheim University Hospital, Trondheim, Norway	Femur	X-ray	-
**Terjesen et al. **[38]** Norway**	63/126	6.6 (3–11) 65%	54, in-toeing gait 5, for hip or thigh pain 4, under control for congenital hip dislocation under treatment	Trondheim University Hospital, Trondheim, Norway	Femur	X-ray	-
**Tomczak et al. **[53]** Germany**	19/37	11 (3.5–17.5) 37%	17, pre-SG assessment for osteotomy 1, only one hip per metal implant in contralateral 1, only CT and US (Perthes and osteomyelitis)	University Hospital of Ulm	Femur	CT, MRI	Intra-observer Inter-observer (*2 observers*)

*CT* computed tomography, *Rx* radiology, *MRI* magnetic resonance imaging, *GM* goniometric, *CP* cerebral palsy, *SG* surgical

All the studies assessed femoral torsion, but only Keppler et al. [52] also included the assessment of tibial torsion. In 7 out of the eight studies, the validity of the methodology with US was analysed: it was compared to CT in 4 studies [47, 51–53], to MRI in 2 studies [51, 53], to X-ray in three studies [38, 49, 50] and to clinical goniometry in one study [51]. Two of the studies analysed the validity by contrasting it with more than one technique [51, 53]. Intra-observer and inter-observer reliability were assessed by 4 of the studies [48, 50, 51, 53].

Four proximal and two distal references were used for femoral assessment. The most used proximal femoral landmarks were the anterior aspect of the femoral neck [48, 50–52] and the anterior tangent between the head and the greater trochanter [38, 49, 53]. For the distal femur, the most used anatomical reference was the posterior tangent of the femoral condyles [49, 52]. However, the most common protocol was to infer the horizontality of these femoral condyles by positioning the leg vertically outside the examination table with the knee flexed 90° [38, 49, 51, 53]. As for the tibial references, the only study included used the posterior tangent of the condyles and the anterior tangent of the distal epiphysis [52] (Table 4).

**Table 4:** Valuation methodology and results

**Study (year) country**	**Valuation methodology with US**		**Results**		**Conclusion**
	Proximal reference	Distal reference	Validity	Reliability	
**Berman et al. **[47]** UK**	POST aspect of FN	Intercondylar axis	7/19 differences $\le$ 5° 4/19 differences 6°–10° 8/19 differences 11°–23°		General use not recommended for FT assessment
**Elke et al. **[50]** Germany**	ANT tangent of FN (horizontal transducer)	Hip with 40° internal rotation	Reproducibility vs Rx 34 hips with wedge at 40° IR: Mean dif = 3.6° *SD* = 3.2° Max dif = 16° 21 hips to 0° legs vertical: Mean dif = 16.6° *SD* = 10.3° Max dif = 33°		The methodology described with IR 40° of hip allows more accurate assessments in high values of femoral torsions
**Gunther et al. **[51]** Germany**	ANT tangent of FN (tilted transducer technique)	Vertical leg (horizontal is assumed as an intercondylar plane)	CT: $\overline{X }$_AVA_ = 34° ± 13.7° / MRI: $\overline{X }$_AVA_ = 23.2° ± 12.8° US: $\overline{X }$_AVA_ = 23.6° ± 8.0° / Clinical A: $\overline{X }$_AVA_ = 27.7° ± 8.5°	INTRA / INTER CT: *r* = 0.99/*r* = 0.96. MRI: *r* = 0.97/*r* = 0.97 US: *r* = 0.88/*r* = 0.88 Clinical A: *r* = 0.47/*r* = 0.77	US and clinical assessments are sufficiently precise for routine assessment. CT and MRI are more accurate for preoperative determination
**Keppler et al. **[52]** Germany**	ANT tangent of FN ANT tangent distal tibial	POST tangent of femoral condyles POST tangent of tibial condyles	FT. CT: $\overline{X }$_AVA_ = 25.2° ± 10.7° / US: $\overline{X }$_AVA_ = 24.7° ± 11.5° (p = 0.42) Comparison CT vs US: Mean dif = -0.5° Max dif = 7° TT. CT: $\overline{X }$_AVA_ = 35.6° ± 10.3° / US: $\overline{X }$_AVA_ = 28.2° ± 9.1° (p = 0.42) Comparison CT vs US: Mean dif = − 1.5° Max dif = 7°	FT. INTER: Mean dif = − 0.10 ± 2.20 Max dif = 6° INTRA. Max dif = 7° *SD* = 1.5° TT. INTER: Mean dif = − 0.9° ± 2.40 Max dif = 7° INTRA: Max dif = 7° *SD* = 1.5°	The methodology presented has a high accuracy of measurement of the length and torsion of lower extremities, is independent of the position of the patient and shows a reliability and reproducibility slightly lower than CT; indicated for both follow-up and surgical planning in adults and children
**Prasad et al. **[48] **UK**	ANT tangent of FN Longitudinal axis of the FN (horizontal probe)	Lateral decubitus (the vertical is assumed as an intercondylar plane)		INTRA / INTER Anterior AVA: ± 6.2° / ± 7.8° True AVA: ± 9.5° / ± 23.5°	The described method of assessment of anterior anteversion has an acceptable level of inter- and intra-observer reliability. The level of inter-observer reliability of the method for the assessment of real anteversion has been unacceptable
**Terjesen and Anda **[49]** Norway**	Groups A and B: ANT tangent of FN Groups C and D: ANT tangent between head and greater femoral trochanter	Groups A and D: vertical leg Groups B and C: posterior tangent femoral condyles	US vs Rx correlation Correlation coefficient in the 4 groups (A–D) were 0.57 to 0.88 1° mean difference between A and B methods Mean difference US vs RX = 4° in C and D groups Difference between US and X-ray < 5° in more than half of the measurements > 10° in 1/10 of measurements		Ultrasound is recommended for screening children with rotational disorders of the femur. In patients with obvious discrepancies between clinical findings and femoral AVA determined by ultrasound, additional radiographic examinations are needed, preferably by CT
**Terjesen et al. **[38]** Norway**	ANT tangent between head and greater femoral trochanter (tilted transducer technique)	Vertical leg (horizontal is assumed as an intercondylar plane)	Correlation coefficient *r* = 0.71 US: $\overline{X }$_AVA_ = 45.3° DE = 5.7° (28°–58°) / Rx: $\overline{X }$_AVA_ = 39.8° *SD* = 8.4° (14°–57°) Consistent difference of 5.5° greater in US Difference increases with age: 3–6 years Mean dif = 4.6° 7–11 years Mean dif = 6.7° Difference between US and Rx $\leq$5° in 63% of measurements > 10° in 6.4% of measurements		US assessment is reliable, fast, painless and safe (non-ionizing). It is recommended for screening for torsional disorders in children. In cases requiring surgery, the evaluation should be complemented with biplanar X-ray or CT to confirm the US data
**Tomczak et al. **[53]** Germany**	ANT tangent between head and greater femoral trochanter (tilted transducer technique)	Vertical leg (horizontal is assumed as an intercondylar plane)	MRI: AVA= $\overline{X }$_AVA_ = 23.2° ± 12.8° (0°–65°) / CT: $\overline{X }$_AVA_ = 34° ± 13.7° (5°–82°) °) US: $\overline{X }$_AVA_ = 25.6° ± 8° (10°–40°) Coef. Pearson: RM vs CT (*r* = 0.77) / RM vs US (**r** = 0.81) / CT vs US (*r* = 0.60)	INTRA and INTER MRI: *r* = 0.97 / *r* = 0.99. TC: *r* = 0.97 / *r* = 0.96 US: *r* = 0.88 / *r* = 0.88	The MRI examination allows a closer approximation to the true AVA compared to US and CT. The acceptable intra- and inter-individual variability of the observer justifies the use of the ultrasound method as screening in children and adolescents. However, a CT or MRI examination should be performed prior to surgical interventions

*POST* posterior, *ANT* anterior, *FN* femoral neck, *FT* femoral torsion, *TT* tibial torsion, *AVA* anteversion angle, *MRI* magnetic resonance imaging, CT tomography computerized, *US* ultrasonography

Both the highest inter- and intra-observer validity and reliability in the assessment of femoral torsion were found using the anterior tangent between the head and the greater trochanter and the posterior tangent of the condyles as references, assuming the horizontality of the condyles with the knees flexed 90° and the legs vertical placed outside the examination Table [38, 49, 53].

The mean differences between US and the gold standard ranged from 4° to 6.7°, and the correlation coefficients ranged from 0.71 to 0.81 [53]. It was not possible to compare the validity data of the assessment of tibial torsion with US, as it was studied in only one article [52]. The highest correlation coefficients for both intra-observer and inter-observer reliability were 0.88 [53] (Table 4).

## Discussion

The data available about the assessment of femoral and/or tibial torsions using US in children have been assessed in terms of validity and reliability, and the proximal and distal anatomical references have been described. Eight studies were considered eligible and were analysed.

### Imaging techniques

All the studies assess the FT, but only one of them, Keppler [52], also analyses TT. Four different imaging techniques were used to assess the validity of US. CT is considered the gold standard for the assessment of torsional disorders [29, 36, 54, 55], and it is the technique used in 4 of the eight studies [47, 51–53]. MRI [51, 53], X-ray [38, 49, 50] and clinical assessment [51] were also used.

A fundamental difference between US and the other imaging tests is that only the surface of mature bone can be observed. This limitation results in differences in the reference planes utilised for US assessments compared to those for CT and MRI, which can capture cross-sectional images to identify epiphyseal planes. In addition, US cannot simultaneously capture both proximal and distal bone landmarks, necessitating separate acquisitions. This can be a limitation if the subject moves between assessments, potentially affecting the results. To address this, simple, secure and comfortable fixation systems can keep the child’s limb immobile on the examination table, preventing any movement between the proximal and distal acquisition [22, 56]. In contrast, CT, MRI and X-ray allow the simultaneous acquisition of both references.

### Anatomical references

#### Proximal femur

Since the US only allows to observe the cortical surface to assess the proximal femur angle, a plane tangent to the bone surface, commonly the anterior plane, is often used. In contrast, the axis of the femoral neck can be used both by CT and MRI. Thus, most studies refer to “true anteversion” as determined by CT and MRI and “anterior anteversion” as determined by US.

Only Prasad et al. [48] assesses the true anteversion by US, since the studied population comprises children aged 0 to 6 months, in which the ossification of the femoral head is non-existent or very incipient, and thus the entire cartilaginous contour can be seen. However, he concludes that the reliability in the assessment of true anteversion with US is not acceptable at this age. Comparing true and anterior anteversion, Terjesen et al. [38] observes a consistent difference of 5° to 10° higher in the latter. Thus, he suggests a correction factor of 5° in children up to 12 years of age and between 5° and 10° in children over 12 years of age and adults.

##### Anatomical references in proximal femur

The anterior anteversion can be determined by different anatomical references, and they may influence the reliability, according to Elke et al. [50]. The references used in the selected studies are the anterior head to the trochanter tangent [38, 49, 53] and the anterior aspect of the femoral neck [48–52]. Elke et al. [50] recommends a degree of inclination of the probe over the anterior proximal femur that allows a correct visualization of the intertrochanteric plane and ensures maximum reliability during the measurement. Alternatively, Terjesen and Anda [49] uses 2 proximal references in his study and concludes that in children it is easier to determine the anterior tangent between the femoral head and the greater trochanter than the anterior tangent of the femoral neck, as the latter is too short. Berman et al. [47], the only author who does not recommend the general use of US for the assessment of FT, uses the posterior aspect of the femoral neck as a reference and finds differences greater than 10° compared to CT in 8 of the 19 femurs analysed. The soft tissues between the probe and the bone and the author’s recognition of some estimation of planes may have influenced the low accuracy of the assessments obtained.

##### Technique with horizontal probe vs inclined probe (tilted transducer technique)

Another feature that influences the reliability is the position of the ultrasound probe on the proximal femur. Berman et al. [47] and Prasad et al. [48] place the probe completely vertically by attaching a spirit level to it. Subsequently, the inclination of the femoral neck is calculated on the image obtained. With this methodology, the accuracy decreases as the torsional value increases, since the lateral area of the femoral neck will move further away from the probe, leading to distortions and measurement errors. To overcome this limitation, Elke et al. [50] proposes a variation of the methodology for large femoral true anteversions: he positions the subject with an internal hip rotation of 40°, thus increasing the parallelism between the femoral neck and the plane of the probe, which is placed completely horizontal, and then, these 40° are added to the value obtained. In his study, Terjesen and Anda [49] proposed a variation of the technique in which the probe is tilted over the proximal femur until it is displayed horizontally on the screen (tilted transducer technique). If an inclinometer is associated with the probe, it will directly provide the degree of inclination of the femoral neck. This is the most widely used technique in subsequent studies.

#### Distal femur

For the distal femur, also different anatomical references have been used: Keppler et al. [52] and Terjesen and Anda [49] used the posterior tangent of the femoral condyles. Gunther et al. [51], Terjesen et al. [38] and Tomczak et al. [53] used a simpler methodology: the horizontal intercondylar plane is inferred using the tibia as a perpendicular reference. This inference is made by flexing the leg vertically beyond the edge of the examination table so that the verticality of the leg assumes the horizontality of the femoral condyles. Prasad et al. [48] assesses infants (0 to 6 months old) by positioning them in lateral decubitus and with the knees flexed 90°, thus, the vertical represents the intercondylar plane. This inference of planes may detract from the validity of the methodology, as the assumed complete planar perpendicularity may not exist. On the other hand, it is much more repeatable, being useful in the monitoring of the same individual, where the initial error in the inference should not vary significantly in successive measurements.

#### Tibial assessment

Out of the selected studies, only Keppler et al. [52] analyses the validity and reliability of the tibial torsion. He uses the posterior tangent of the tibial condyles and the tangent of the distal anterior tibial face as references. Similar to the proximal femur, some of the reference axes used by CT and MRI to determine tibial torsion, are not detectable by US, for example the intermalleolar axis.

Several studies evaluate US in the assessment of tibial torsion [14, 21, 22, 39], but to the best of the authors’ knowledge, no study has analysed its reliability and validity in a paediatric population without neurological alterations.

The anatomical references used by the authors for the assessment of femoral and tibial torsion are listed in Table 4.

### Validity of US vs other imaging tests

Regarding the imaging tests used to analyse the validity of the US, Berman et al. [47] and Keppler et al. [52] compare it with TC, Gunther et al. [51] and Tomczak et al. [53] compare to CT and MRI, and Elke et al. [50] and Terjesen [38, 49] use biplanar radiography.

Gunther et al. [51]*,* Terjesen [38, 49] and Tomczak et al. [53] conclude that US is a good alternative, endowed with sufficient precision for routine clinical examinations and monitoring of FT in children. However, for the preoperative planning of torsional disorders, the use of CT, MRI or biplanar radiography is preferable due to their higher accuracy. One of the major drawbacks of US assessment of femoral and tibial torsion is that both ends of the bony structure are not visualised simultaneously. Thus, those methodologies that use both proximal and distal reference to determine torsion require the subject to remain completely immobile during the assessment [47, 49, 52]. Keppler et al. [52] discusses a method of assessment with US that incorporates markers on the probe and 3D reference systems, which eliminates dependence during assessment on the subject’s position or movements. This allows him to also indicate US for pre-surgical assessment of both FT and TT. Only Berman et al. [47] does not recommend the general use of US for FT assessment due to the low validity results obtained. To be noted, his study was conducted in 1987; the evolution of US apparatus and the protocols may have increased the validity of the assessment.

### Evaluation of inter- and intra-observer reliability

Inter-observer and intra-observer reliability have been analysed by 4 out of the 8 studies selected [48, 51–53]. The results were considered good for FT. Gunther et al. [51] and Tomczak et al. [53] obtain identical reliability data for both interobserver (*r* = 0.88) and intra-observer (*r* = 0.88). These results are good but lower than the reliability obtained with CT and MRI, with *r* values greater than 0.95. Furthermore, Gunther et al. [51] also compares it with the reliability of the clinical assessment, which obtains low inter- and intra-observer values; *r* = 0.47 and *r* = 0.77, respectively. In the Prasad infant study [48], the reliability of FT assessment with US using anterior anteversion as a proximal reference was clearly superior to the true anteversion. US observation of the entire cross-section of the head and trochanter in infants is unclear, given the lack of sharpness of the posterior face. This leads to an inaccurate determination of the real axis of the femoral neck and a low reproducibility.

Keppler et al. [52] incorporates a 3D reference system to the US that allows a non-motion-dependent assessment of the patient during acquisition. This method obtains better reliability results to CT for both femoral torsion and tibial torsion.

Most of the studies analysed conclude that US is a reliable method for the assessment and follow-up of femoral torsional alterations in children. It is useful due to its immediacy, convenience and safety, as it does not expose the children to ionising radiation. However, the validity and reliability data obtained by MRI, CT and biplanar X-ray are usually superior to US. Thus, MRI and biplanar X-ray would be the methods of choice in the surgical planning of femoral torsional alterations in children, due to their null or low exposure to ionising radiation, respectively. Despite being considered the gold standard, the use of CT is not recommended for follow-up in children due to the high exposure to ionising radiation it represents. Data for TT are only available from the Keppler study [52].

The method proposed by Keppler [52] obtains excellent results in terms of both validity and reliability, only slightly lower than those obtained with CT. Thus, he is the only author who validated US not only for the follow-up of torsional alterations of the lower extremity but also for surgical planning in adults and children. Noteworthy, this study is the only one in which a 3D reference system is used, clearly increasing them.

While some studies report good reliability for femoral torsion, particularly in paediatric populations, there is limited data available for tibial torsion assessment without neurological alterations. Future research should address this gap to provide a more comprehensive understanding of the reliability of US in assessing lower extremity torsional abnormalities in paediatric population.

## Strengths and limitations

In this systematic review, all articles were included based on the selection criteria, without any restrictions on the year or language of publication. Except for the paper by Prasad et al., all other articles belong to the last century. This trend highlights the paucity of recent studies addressing the validity and reliability of US in evaluating lower extremity torsional abnormalities in the paediatric and adolescent population, particularly in relation to tibial torsion. Additionally, the age of the selected articles contributes to the observation that many of them present outcome analysis strategies that do not align with current standards.

To improve the precision and effectiveness of our review, we have revised the protocol originally registered with the PROSPERO Systematic Reviews database under ID CRD42021290973. While the initial protocol included participants of all ages to ensure a comprehensive search scope, it primarily aimed to examine children and adolescents. To better align with our research goals, the revised protocol now specifically targets this younger demographic. Furthermore, we have replaced the Downs & Black checklist with the Quadas & Stard protocol by Whiting et al. [44], which better suits the types of studies under consideration.

## Conclusions

All the studies included in this systematic review assessed femoral torsion, but only one of them also included the assessment of tibial torsion. Seven out of the eight studies evaluated the validity of the ultrasound (US) methodology by comparing it with CT, MRI, X-ray or clinical goniometry. Four studies examined intra-observer and inter-observer reliability.

The validity and reliability of US in the assessment of lower limb torsions can be classified from acceptable to high depending on the measurement protocol and the anatomical references used.

US has proven to be a valuable tool in the routine assessment and follow-up of femoral torsional alterations in children and adolescent. Its advantages lie in its safety, cost-effectiveness and the immediacy of results, which can be particularly beneficial in a clinical setting where timely diagnosis and treatment are crucial.

However, while US is accurate and reliable enough for clinical assessment of lower extremity torsion disorders, its suitability for surgical planning is controversial. Three authors recommend more accurate imaging techniques, such as MRI and biplanar radiography. Only one author, who used a 3D reference system, achieved sufficiently precise results to consider US a suitable technique for surgical planning.

These findings have significant clinical implications. They suggest a need for a multi-modal imaging approach in managing femoral torsional disorders in children, with US serving as a first-line tool for initial assessment and follow-up and MRI or biplanar radiography being reserved for pre-surgical planning.

Finally, this systematic review highlights the lack of recent studies analysing the validity and reliability of US for assessing tibial and femoral torsion in children and adolescents, especially TT. It is particularly significant in a technique that uses devices that have evolved largely along these years. This underlines an area for future research, which could potentially lead to improved diagnostic and treatment strategies for this patient population.

## Data Availability

The datasets used and/or analysed during the present study can be requested from the corresponding author.

## Abbreviations

ANT: Anterior
AV: Anteversion
AVA: Anteversion angle
CP: Cerebral palsy
CT: Computer tomography
FN: Femoral neck
FT: Femoral torsion
GM: Goniometric
MRI: Magnetic resonance imaging
POST: Posterior
Rx: Radiology
SG: Surgical
US: Ultrasonography

## References

[1] Fabry G (2010) Clinical practice: static, axial, and rotational deformities of the lower extremities in children. Eur J Pediatr 169(5):529–534 20052491 10.1007/s00431-009-1122-x

[2] Weinberg DS, Park PJ, Morris WZ, Liu RW (2015) Femoral version and tibial torsion are not associated with hip or knee arthritis.pdf. J Pediatr Orthop 00(00):e120-8 10.1097/BPO.0000000000000604 26214325

[3] Kraeutler MJ, Chadayammuri V, Garabekyan T, Mei-Dan O (2018) Femoral version abnormalities significantly outweigh effect of Cam Impingement on Hip Internal Rotation. J Bone Joint Surg Am 100(3):205–210 29406341 10.2106/JBJS.17.00376

[4] Li H, Wang Y, Oni JK, Qu X, Li T, Zeng Y, Liu F, Zhu Z (2014) The role of femoral neck anteversion in the development of osteoarthritis in dysplastic hips. Bone Joint J 96-B(12):1586–93 25452359 10.1302/0301-620X.96B12.33983

[5] Kaiser P, Schmoelz W, Schoettle P, Zwierzina M, Heinrichs C, Attal R (2017) Increased internal femoral torsion can be regarded as a risk factor for patellar instability - a biomechanical study. Clin Biomech (Bristol, Avon) 47:103–109 28628800 10.1016/j.clinbiomech.2017.06.007

[6] Ahn JK, Kwon DR, Park GY, Lee KH, Rim JH, Bin Jung W et al (2017) Therapeutic effect of microcurrent therapy in children with in-toeing gait caused by increased femoral anteversion: a pilot study. Ann Rehabil Med 41(1):104–12 28289642 10.5535/arm.2017.41.1.104 PMC5344811

[7] Sass P, Hassan G (2003) Lower extremity abnormalities in children. Am Fam Physician 68(3):461–468 12924829

[8] Rosselli Cock P, Duplat Lapides JL (2012) Ortopedia infantil. 2nd. Ed. Colombia: Médica Panamericana

[9] Staheli LT (2016) Fundamentos de ortopedia pedit̀rica/Fundamentals of pediatric orthopedics. 5^th^. Ed. Lippincott Williams & Wilkins

[10] Yoon TL, Park KM, Choi SA, Lee JH, Jeong HJ, Cynn HS (2014) A comparison of the reliability of the trochanteric prominence angle test and the alternative method in healthy subjects. Man Ther 19(2):97–101 24035201 10.1016/j.math.2013.07.011

[11] Sangeux M, Mahy J, Graham HK (2014) Do physical examination and CT-scan measures of femoral neck anteversion and tibial torsion relate to each other? Gait Posture 39(1) 10.1016/j.gaitpost.2013.05.020 23787150

[12] Ruwe PA, Gage JR, Ozonoff MB, DeLuca PA (1992) Clinical determination of femoral anteversion. A comparison with established techniques. J Bone Joint Surg Am 74(6):820–30 1634572

[13] Milner CE, Soames RW (1998) A comparison of four in vivo methods of measuring tibial torsion. J Anat [Internet] 193(1):139–144 9758144 10.1046/j.1469-7580.1998.19310139.x PMC1467830

[14] Butler-Manuel PA, Guy RL, Heatley FW (1992) Measurement of tibial torsion - a new technique applicable to ultrasound and computed-tomography. Br J Radiol 65(770):119–126 1540801 10.1259/0007-1285-65-770-119

[15] Tamari K, Tinley P (2003) A new concept of estimating tibiofibular torsion: an in vivo reliability study. J Orthop Sports Phys Ther 33(2):85–90 12619747 10.2519/jospt.2003.33.2.85

[16] Davids JR, Benfanti P, Blackhurst DW, Allen BL (2002) Assessment of femoral anteversion in children with cerebral palsy: accuracy of the trochanteric prominence angle test. J Pediatr Orthop 22(2):173–178 11856924

[17] Staheli LT, Corbett M, Wyss C, King H (1985) Lower-extremity rotational problems in children. Normal values to guide management. J Bone Joint Surg Am 67(1):39–47 3968103

[18] Gulan G, Matovinović D, Nemec B, Rubinić D, Ravlić-Gulan J (2000) Femoral neck anteversion: values, development, measurement, common problems. Coll Antropol 24(2):521–527 11216420

[19] MacWilliams BA, McMulkin ML, Davis RB, Westberry DE, Baird GO, Stevens PM (2016) Biomechanical changes associated with femoral derotational osteotomy. Gait Posture 49:202–206 27450671 10.1016/j.gaitpost.2016.07.002

[20] Karam M, Bizdikian AJ, Khalil N, Bakouny Z, Obeid I, Ghanimeh J et al (2020) Alterations of 3D acetabular and lower limb parameters in adolescent idiopathic scoliosis. Eur Spine J 29(8):2010–2017 32246232 10.1007/s00586-020-06397-5

[21] Joseph B, Carver RA, Bell MJ, Sharrard WJ, Levick RK, Aithal V et al (1987) Measurement of tibial torsion by ultrasound. J Pediatr Orthop 7(3):317–323 3294897 10.1097/01241398-198705000-00014

[22] Hudson D, Royer T, Richards J (2006) Ultrasound measurements of torsions in the tibia and femur. J Bone Joint Surg Am 88(1):138–143 16391259 10.2106/JBJS.D.02924

[23] Davids JR, Davis RB (2007) Tibial torsion: significance and measurement. Gait Posture 26(2):169–171 17544274 10.1016/j.gaitpost.2007.05.002

[24] Rosskopf AB, Buck FM, Pfirrmann CWA, Ramseier LE (2017) Femoral and tibial torsion measurements in children and adolescents: comparison of MRI and 3D models based on low-dose biplanar radiographs. Skeletal Radiol 46(4):469–476 28154901 10.1007/s00256-017-2569-x

[25] Schmaranzer F, Lerch TD, Siebenrock KA, Tannast M, Steppacher SD (2019) Differences in femoral torsion among various measurement methods increase in hips with excessive femoral torsion. Clin Orthop Relat Res 477(5):1073–1083 30624313 10.1097/CORR.0000000000000610 PMC6494336

[26] Haller TV, Schenk P, Jud L, Hoch A, Goetschi T, Zingg PO (2021) Consistency of 3D femoral torsion measurement from MRI compared to CT gold standard. BMC Musculoskelet Disord 22(1) 10.1186/s12891-021-04633-7 PMC8403345 34454445

[27] Güven M, Akman B, Ünay K, Özturan EK, Çakıcı H, Eren A (2009) A new radiographic measurement method for evaluation of tibial torsion: a pilot study in adults. Clin Orthop Relat Res 467(7):1807–1812 19052824 10.1007/s11999-008-0655-z PMC2690742

[28] Gheno R, Nectoux E, Herbaux B, Baldisserotto M, Glock L, Cotten A, Boutry N (2012) Three-dimensional measurements of the lower extremity in children and adolescents using a low-dose biplanar X-ray device. Eur Radiol 22(4):765–771 22011904 10.1007/s00330-011-2308-y

[29] Botser IB, Ozoude GC, Martin DE, Siddiqi AJ, Kuppuswami S, Domb BG (2012) Femoral anteversion in the hip: comparison of measurement by computed tomography, magnetic resonance imaging, and physical examination. Arthroscopy 28(5):619–627 22301362 10.1016/j.arthro.2011.10.021

[30] Koenig JK, Pring ME, Dwek JR (2012) MR evaluation of femoral neck version and tibial torsion. Pediatr Radiol 42(1):113–115 21842328 10.1007/s00247-011-2206-0

[31] Muhamad AR, Freitas JM, Bomar JD, Dwek J, Hosalkar HS (2012) CT and MRI lower extremity torsional profile studies: measurement reproducibility. J Child Orthop 6(5):391–396 24082954 10.1007/s11832-012-0434-y PMC3468734

[32] Hesham K, Carry PM, Freese K, Kestel L, Stewart JR, Adam Delavan J et al (2017) Measurement of femoral version by MRI is as reliable and reproducible as CT in children and adolescents with hip disorders. J Pediatr Orthop 37(8):557–562 28323254 10.1097/BPO.0000000000000712 PMC5368029

[33] Chaibi Y, Cresson T, Aubert B, Hausselle J, Neyret P, Hauger O et al (2012) Fast 3D reconstruction of the lower limb using a parametric model and statistical inferences and clinical measurements calculation from biplanar X-rays. Comput Methods Biomech Biomed Engin 15(5):457–466 21229412 10.1080/10255842.2010.540758

[34] Guenoun B, Zadegan F, Aim F, Hannouche D, Nizard R (2012) Reliability of a new method for lower-extremity measurements based on stereoradiographic three-dimensional reconstruction. Orthop Traumatol Surg Res 98(5):506–513 22858107 10.1016/j.otsr.2012.03.014

[35] Gaumetou E, Quijano S, Ilharreborde B, Presedo A, Thoreux P, Mazda K et al (2014) EOS analysis of lower extremity segmental torsion in children and young adults. Orthop Traumatol Surg Res 100(1):147–151 24439563 10.1016/j.otsr.2013.09.010

[36] Folinais D, Thelen P, Delin C, Radier C, Catonne Y, Lazennec JY (2013) Measuring femoral and rotational alignment: EOS system versus computed tomography. Orthop Traumatol Surg Res 99(5):509–516 23877073 10.1016/j.otsr.2012.12.023

[37] Takeuchi S, Goto H, Iguchi H, Watanabe N, Osaga S, Murakami H et al (2019) Ultrasonographic assessment of femoral torsion angle based on tilting angles of femoral neck and condylar axis. Ultrasound Med Biol 45(8):1970–1976 31064699 10.1016/j.ultrasmedbio.2019.03.022

[38] Terjesen T, Anda S, Rønningen H (1993) Ultrasound examination for measurement of femoral anteversion in children. Skeletal Radiol 22(1):33–36 8430343 10.1007/BF00191522

[39] Hudson D (2008) A comparison of ultrasound to goniometric and inclinometer measurements of torsion in the tibia and femur. Gait Posture 28(4):708–710 18555685 10.1016/j.gaitpost.2008.04.017

[40] Hafiz E, Hiller CE, Nicholson LL, Nightingale EJ, Clarke JL, Grimaldi A et al (2014) Development of a method for measuring femoral torsion using real-time ultrasound. Physiol Meas 35(7):1335–1348 24854205 10.1088/0967-3334/35/7/1335

[41] Kulig K, Harper-Hanigan K, Souza RB, Powers CM (2010) Measurement of femoral torsion by ultrasound and magnetic resonance imaging: concurrent validity. Phys Ther 90(11):1641–1648 20724419 10.2522/ptj.20090391

[42] Page MJ, McKenzie JE, Bossuyt PM, Boutron I, Hoffmann TC, Mulrow CD et al (2021) The PRISMA 2020 statement: an updated guideline for reporting systematic reviews, vol 372. BMJ Publishing Group, The BMJ 10.1136/bmj.n71 PMC8005924 33782057

[43] Bossuyt PM, Reitsma JB, Bruns DE, Gatsonis CA, Glasziou PP, Irwig LM, Moher D, Rennie D, de Vet HC, Lijmer JG (2003) Standards for reporting of diagnostic accuracy. The STARD statement for reporting studies of diagnostic accuracy: explanation and elaboration. Ann Intern Med 138(1):W1-12 12513067 10.7326/0003-4819-138-1-200301070-00012-w1

[44] Whiting PF, Weswood ME, Rutjes AW, Reitsma JB, Bossuyt PN, Kleijnen J (2006) Evaluation of QUADAS, a tool for the quality assessment of diagnostic accuracy studies. BMC Med Res Methodol 6(6):9 16519814 10.1186/1471-2288-6-9 PMC1421422

[45] Fernandes De Oliveira MR, Gomes A de C, Toscano CM (2011) Almério de Castro Gomes II QUADAS and STARD: evaluating the quality of diagnostic accuracy studies. Rev Saúde Pública 45(2) 10.1590/s0034-89102011000200021 21412577

[46] Lutsenko MA (2023) Assessing measurement quality: as a unifying and consistent. Monarch Management Review 2(1)

[47] Berman L, Mitchell R, Katz D (1987) Ultrasound assessment of femoral anteversion. a comparison with computerised tomography. J Bone Joint Surg Br 69(2):268–70 3546330 10.1302/0301-620X.69B2.3546330

[48] Prasad SS, Bruce C, Crawford S, Higham J, Garg N (2003) Femoral anteversion in infants: a method using ultrasound. Skeletal Radiol 32(8):462–467 12845491 10.1007/s00256-003-0652-y

[49] Terjesen T, Anda S (1987) Femoral anteversion in children measured by ultrasound. Acta Orthop Scand 58(4):403–407 3314318 10.3109/17453678709146366

[50] Elke R, Ebneter A, Dick W, Fliegel C, Morscher E (1991) Die sonographische Messung der Schenkelhalsantetorsion. Z Orthop Ihre Grenzgeb 129(02):156–163 1829297 10.1055/s-2008-1040176

[51] Gunther KP, Kessler S, Tomczak R, Pfeifer P, Puhl W (1996) Femoral anteversion - reliability and clinical significance of different investigation techniques in children and adolescents. Z Orthop Ihre Grenzgeb 134(4):295–301 8928555 10.1055/s-2008-1039764

[52] Keppler P, Strecker W, Kinzl L, Simmnacher M, Claes L (1999) Determination of leg geometry by ultrasound. Orthopade 28(12):1015–1022 10672602 10.1007/s001320050427

[53] Tomczak R, Gunther K, Pfeifer T, Haberle HJ, Rieber A, Danz B et al (1995) Measurement of anteversion of the femoral neck in children by MRI and evaluation by comparison with CT and ultrasound. RoeFo 162(3):224–228 10.1055/s-2007-1015869 7718777

[54] Arazi M, Öǧün TC, Memik R (2001) Normal development of the tibiofemoral angle in children: a clinical study of 590 normal subjects from 3 to 17 years of age. J Pediatr Orthop 21(2):264–267 11242264

[55] Sugano N, Noble PC, Kamaric E (1998) A comparison of alternative methods of measuring femoral anteversion. J Comput Assist Tomogr 22(4):610–4 9676454 10.1097/00004728-199807000-00019

[56] Tamari K, Tinley P, Briffa K, Breidahl W (2005) Validity and reliability of existing and modified clinical methods of measuring femoral and tibiofibular torsion in healthy subjects: use of different reference axes may improve reliability. Clin Anat 18(1):46–55 15597368 10.1002/ca.20050

